# Map of open and closed chromatin domains in Drosophila genome

**DOI:** 10.1186/1471-2164-15-988

**Published:** 2014-11-18

**Authors:** Beatrice Milon, Yezhou Sun, Weizhong Chang, Todd Creasy, Anup Mahurkar, Amol Shetty, Dmitry Nurminsky, Maria Nurminskaya

**Affiliations:** Department of Biochemistry and Molecular Biology, School of Medicine, University of Maryland, 108 N. Greene St., Baltimore, MD 21201 USA; Institute for Genome Sciences, School of Medicine, University of Maryland, Baltimore, MD 21201 USA

**Keywords:** Chromatin structure, Nuclease, Gene expression, Chromatin modifications

## Abstract

**Background:**

Chromatin compactness has been considered a major determinant of gene activity and has been associated with specific chromatin modifications in studies on a few individual genetic loci. At the same time, genome-wide patterns of open and closed chromatin have been understudied, and are at present largely predicted from chromatin modification and gene expression data. However the universal applicability of such predictions is not self-evident, and requires experimental verification.

**Results:**

We developed and implemented a high-throughput analysis for general chromatin sensitivity to DNase I which provides a comprehensive epigenomic assessment in a single assay. Contiguous domains of open and closed chromatin were identified by computational analysis of the data, and correlated to other genome annotations including predicted chromatin “states”, individual chromatin modifications, nuclear lamina interactions, and gene expression. While showing that the widely trusted predictions of chromatin structure are correct in the majority of cases, we detected diverse “exceptions” from the conventional rules. We found a profound paucity of chromatin modifications in a major fraction of closed chromatin, and identified a number of loci where chromatin configuration is opposite to that expected from modification and gene expression patterns. Further, we observed that chromatin of large introns tends to be closed even when the genes are expressed, and that a significant proportion of active genes including their promoters are located in closed chromatin.

**Conclusions:**

These findings reveal limitations of the existing predictive models, indicate novel mechanisms of epigenetic regulation, and provide important insights into genome organization and function.

**Electronic supplementary material:**

The online version of this article (doi:10.1186/1471-2164-15-988) contains supplementary material, which is available to authorized users.

## Background

Chromatin compactness is the key feature of chromatin that reflects its accessibility to transcription machinery. Tightly packed closed chromatin is considered a hallmark of gene silencing, and chromatin opening precedes lineage-specific gene expression thus providing an excellent indicator of cell fate commitment [1, 2]. However, genome-wide analyses of chromatin configuration have been focused not on direct assessment of chromatin compactness, but on predictions based on chromatin marks such as DNA methylation and histone modifications. Predictive models recognize numerous chromatin “states” presumably indicating regulatory elements, gene activity, and other aspects of genome biology [3, 4], thereby deducing chromatin configuration from our knowledge of gene expression and chromatin marks. Such predictive approach intrinsically limits the discovery of novel mechanistic links between chromatin configuration and gene expression as well as chromatin modifications, necessitating development of the alternative, more direct means to analyze genome-wide patterns of open and closed chromatin. Moreover, although the models of predicted “states” are excellent tools for basic research, they require examination of numerous chromatin marks in multiplicity of assays and thus are not readily applicable to routine analysis of small clinical samples.

**Figure 1:**
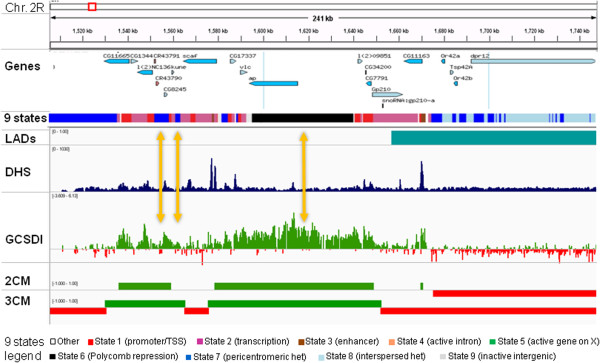
**Genome viewer snapshot shows domains of open and closed chromatin detected by GCSDI.** An approximately 250 kbp window demonstrates the relationship of differential GCSDI signal, 2CM, and 3CM models to the genes, predicted 9 chromatin states, and lamina-associated domains (LADs). DNase I hypersensitive site assay signal (DHS) is shown for comparison. Open chromatin domains are shown in green and closed – in red in 2CM and 3CM traces. Discrepancies between chromatin structure predictions (repressed) and chromatin compactness (open) are outlined by arrows.

Here, we sought clarification on two important topics in chromatin biology: first, whether there is a straightforward and immutable association between chromatin compactness and certain chromatin modifications or combinations thereof, and second – what is the relation between chromatin compactness and gene expression. Toward this objective we designed and validated a high throughput assay for general chromatin sensitivity to DNase I (GCSDI) as a powerful approach to determine chromatin compactness across genome. Previous low-throughput studies have established the power of GCSDI to detect domains of both open and closed chromatin in specific genomic loci [5–8]. This feature separates GCSDI from other current experimental approaches to chromatin structure, which employ nuclease or transposase treatment to detect unusually open chromatin regions (e.g. hypersensitive sites) and/or nucleosome positioning [9–11], but do not reveal contiguous open and closed chromatin domains that have been broadly implicated in gene regulation. Specifically, the difference between GCSDI and the popular analysis for DNase I hypersensitive sites (DHS) is that while both methods use DNase I to introduce single-stranded nicks in accessible DNA, DHS detects only sites where density of nicks is high enough to generate double-stranded DNA breaks, while GCSDI analyzes frequency of nicks continuously across the region of interest (and hypersensitive sites appear as signal peaks in open chromatin domains, such as shown in Figure 1). Capitalizing on excellent annotation of *Drosophila* genome and on previous genome-wide analyses of chromatin marks in *Drosophila* Schneider-2 (*S2*) cell line, we have created the map of chromatin compactness in these cells and cross-referenced it to known patterns of chromatin modifications and gene expression.

## Results and discussion

To measure GCSDI across genome we combined a brief DNase I treatment of permeabilized cells with random amplification of the DNase I-nicked genomic DNA, followed with analysis of sequence representation in amplified material by a high-throughput method. DNase I preferentially nicks DNA in open chromatin, rendering these regions inefficient template for amplification and thus predisposing them for under-representation in amplified material. The difference in representation between known open and closed chromatin loci can be reliably detected by the GCSDI assay after treatment with diverse amounts of DNase I, and thus is a reliable analysis outcome that is not overly sensitive to DNase I treatment conditions (Additional file 1: Figure S1).

To generate a GCSDI profile across genome, amplified DNA samples from DNase I-treated and control untreated cells (n = 2) were hybridized with tiling Affymetrix microarrays, signal intensities for each probe were averaged within the experimental groups and fold differences between the groups were calculated. Positive log2 values were assigned to the sequences underrepresented in DNase I-treated sample (open) and negative values - to the sequences overrepresented in DNase I-treated sample (closed). The identified open and closed chromatin regions were extensive and contiguous, consistent with previous low-throughput studies [5–8] and in contrast with the narrow discrete regions detected by the DHS assay [4, 12] (Figure 1, GCSDI versus DHSs tracks).

Two segmentation models of chromatin compactness were created using the genome-wide GCSDI profiles. (i) We used a sliding window algorithm to identify transition points and to segment genome into contiguous series of open or closed chromatin domains, with the mean size of 15 kb and ranging up to 500 kb (Additional file 2: Figure S2A). Resulting Two-Configuration Model (referred hereafter as 2CM) is well compatible with other large-scale genome features such as lamina-associated domains (LADs) [10] but did not provide sufficient resolution for analysis of some gene-dense regions with small genes. This problem was overcome by implementing another type of analysis (ii) using HMM to identify positive or negative peaks of differential signals, and consolidating clusters of such peaks into domains. This approach to identification of closed and open chromatin domains was more selective, but at a cost of assigning about one-third of genome to domains that are neither open nor closed, thereby defined as “neutral”. Thus, the outcome of such analysis was a Three-Configuration Model (3CM) of domains with the mean size of 3–10 kb (Additional file 2: Figure S2B). Further analyses showed similar results for 2CM and 3CM. We present findings for 2CM in the main figures and the majority of results for 3CM, are shown in Additional file figures.

**Figure 2:**
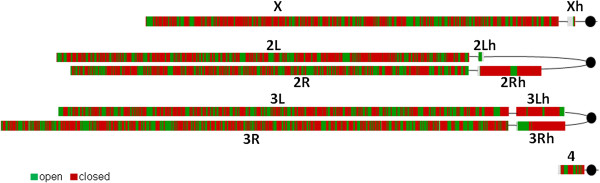
**Distribution of open (green) and closed (red) chromatin domains detected by 2CM analysis on chromosomes of** ***D. melanogaster*** **.**

**Figure 3:**
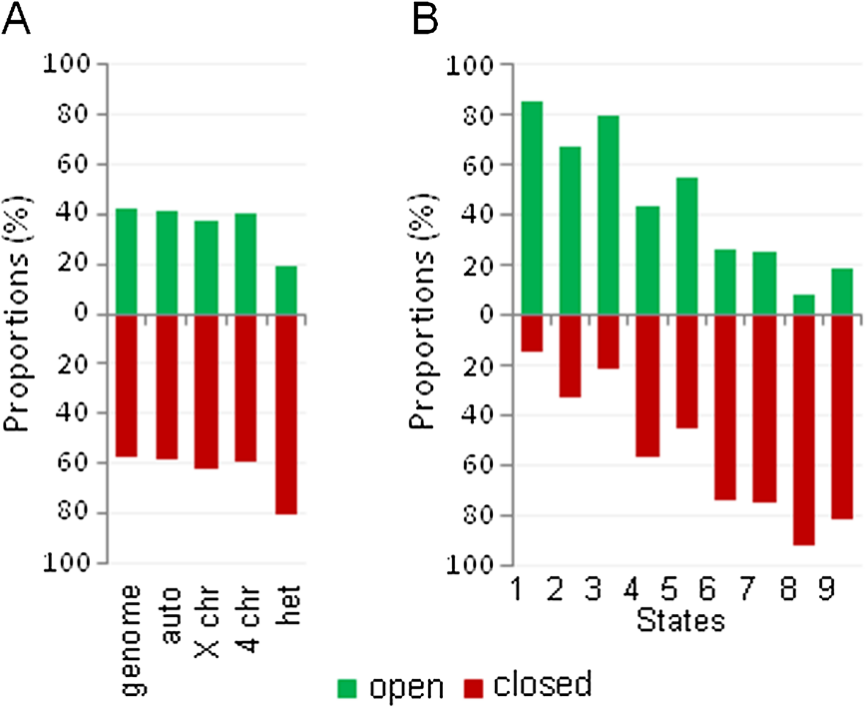
**Representation of detected open and closed chromatin domains in genome and their association with predicted chromatin states.** Proportions of open and closed chromatin detected by 2CM are shown for individual chromosomes **(A)** and for the genome regions predicted as 9 chromatin states [4]**(B)**.

Domains of open, closed, and neutral chromatin identified by both models appeared interspersed across genome (Figure 2, Additional file 3: Figure S3). In euchromatin, 2CM detected approximately 60% of genome in closed chromatin and 40% - in open (Figure 3A), while 3CM detected 37% of genome in closed, 23% in open, and 40% in neutral chromatin (Additional file 4: Figure S4A). Both the pericentromeric heterochromatin regions and chromosome 4 were heavily enriched with neutral chromatin in 3CM, however heterochromatin only (not chromosome 4) showed an overabundance of closed chromatin in 2CM. Therefore, chromosome 4 appears to share overall similarities with both euchromatin and heterochromatin, consistent with known interspersion of unique sequences and repeat clusters in this genome region [13].

**Figure 4:**
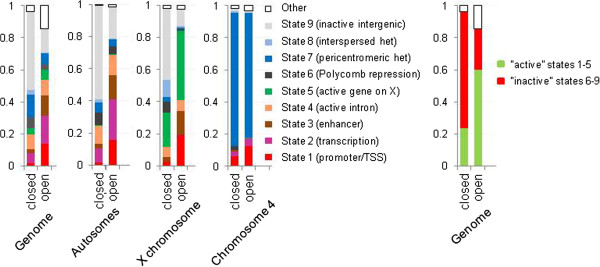
**Contributions of the predicted chromatin states to open and closed chromatin.** Results are shown for the whole genome, and separately for major autosomes and chromosomes X and 4 (left panels). In addition, cumulative contribution of the states 1–5 considered “active” chromatin, and of the states 6–9 considered “inactive” or repressed, are shown for open and closed chromatin in the whole genome (right panel).

Next, we analyzed the relationships between identified open and closed chromatin domains and chromatin modifications, beginning with the nine major chromatin predicted “states” [4]. In general, predictions were confirmed in agreement with previous research linking chromatin opening with cis-regulation and gene expression [5–9] as states 1 through 3 (regulatory and transcribed sequences) mostly corresponded to the open chromatin and the states 6, 8, and 9 (Polycomb-mediated repression, intercalated heterochromatin, and silent intergenic regions) were predominantly identified as closed (Figure 3B, Additional file 4: Figure S4B). State 7 (pericentromeric heterochromatin) was mostly identified as closed in 2CM and neutral in 3CM consistent with enrichment of the pericentromeric regions with these configurations (Figure 2, Additional file 3: Figure S3). In a complementary analysis, we found that a major proportion of open chromatin has been predicted as one of the “active” chromatin states 1 through 3, and the majority of closed chromatin - as “inactive” states 6 through 9 (Figure 4, Additional file 5: Figure S5). Although the state 5 (active genes on the X chromosome) appeared to be similarly represented by open and closed chromatin in the whole-genome study, analysis focusing on the X chromosome showed this state representing about 40% of open and a lesser fraction of closed chromatin. Thus, overall results of our analysis of chromatin compactness were consistent with the chromatin state predictions based on chromatin modification marks, providing cross-validation of these two approaches. However there were a number of discrepancies as well. We were unable to find specific correlations between chromatin compactness and state 4 (active gene introns) which was equally distributed between open and closed chromatin and contributed about 10% to all chromatin configurations (Figures 3 and 4, Additional file 4: Figure S4 and Additional file 5: Figure S5); noteworthy, this promiscuous distribution pertained to all four distinct sub-states 18, 19, 20 and 21 which have been consolidated in state 4 [4] (Additional file 4: Figure S4C). This finding reflected a peculiar relationship between gene expression and intron chromatin structure, described in more detail below. Also, a relatively minor portion (23%) of the closed chromatin detected in our genome-wide analysis has been predicted as “active” states 1 through 3 and a similar fraction of open chromatin (26%) – as “inactive” states 6 through 9 (Figure 4). A visual inspection of the GCSDI signal distribution showed that at least some of these mismatches represented genuine differences between the direct and the predictive approaches to chromatin structure analysis. Figure 1 provides an example: yellow arrows indicate open chromatin detected in the regions predicted as “heterochromatin” (blue) and “Polycomb-repressed” (black). These findings identify cases of potentially novel unconventional epigenetic regulation which warrant further mechanistic inquiries.

**Figure 5:**
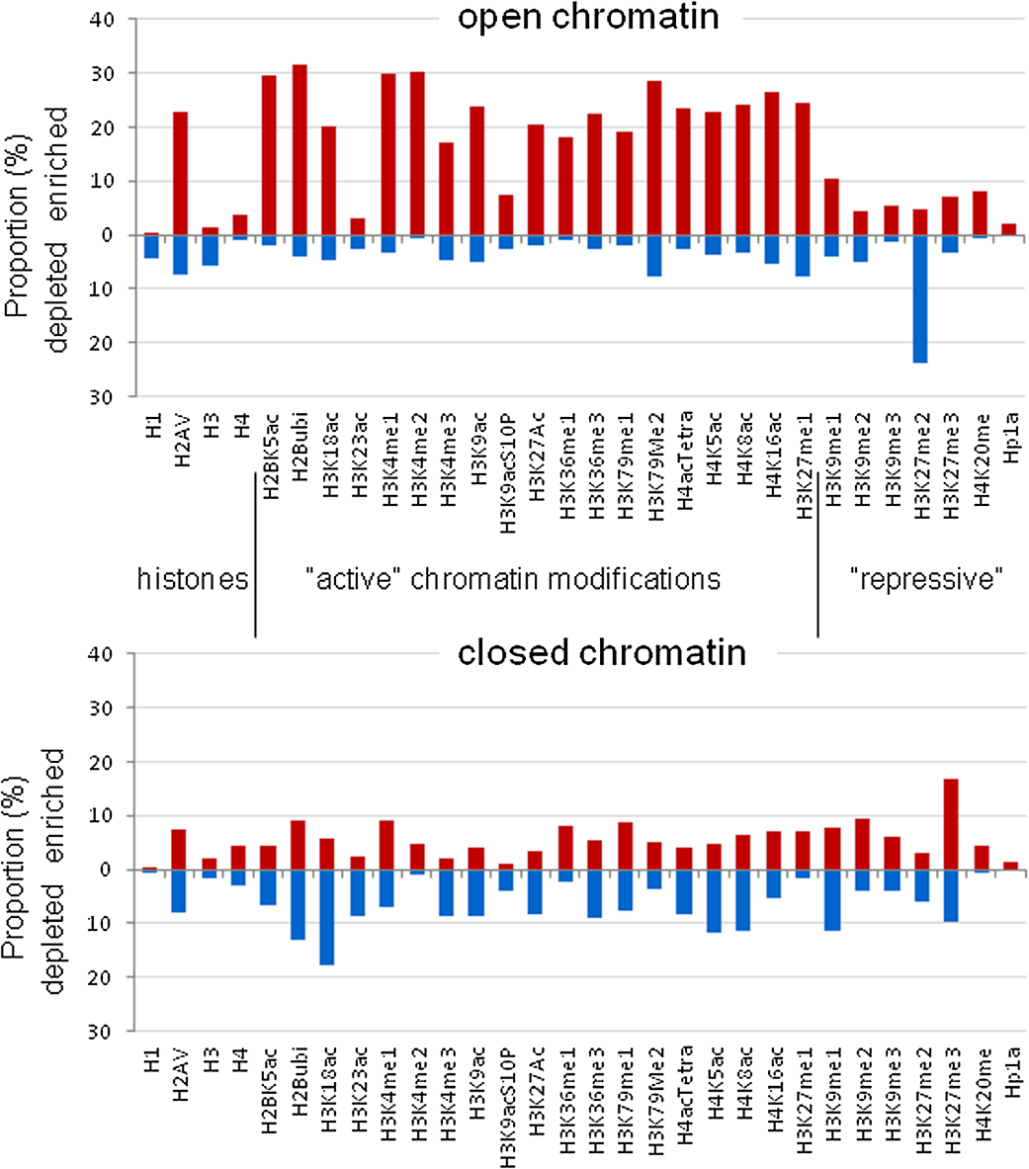
**Enrichment and depletion of chromatin modifications in open and closed chromatin.** Bars show percent proportions of regions enriched with (red) or depleted of (blue) particular chromatin modifications in open and closed chromatin domains detected by 3CM. Data are cumulative for the entire genome.

We also analyzed distribution of individual modifications which have been traditionally linked to certain chromatin structure predictions, and intriguingly found that while open chromatin was associated with numerous abundant chromatin modifications, closed chromatin was largely unmodified. This was true in whole-genome analysis (Figure 5) and also when the major autosome euchromatin, X chromosome, heterochromatin, and chromosome 4 were analyzed separately (Additional file 6: Figure S6). Histone acetylation (with single exception of H3K23), ubiquitination, phosphorylation, and methylation at H3K4, H3K36, and H3K79 were indicators of open chromatin, and depletion of these modifications was characteristic of closed domains. Among the positive indicators of closed chromatin, dimethylation of H3K9 and especially trimethylation of H3K27 were prominent, but still enrichment with these modifications accounted only for less than one-quarter of closed chromatin in 2CM and one-third in 3CM analysis. Thus, the prevalent mechanisms underlying chromatin closing do not appear to extensively rely on known chromatin marks, indicating that yet unknown chromatin compaction-related modifications may exist - or perhaps that “closed” is the default state of unmodified chromatin (note that abundant DNA methylation is lacking in *Drosophila*, hence it has little direct contribution to chromatin structure).

**Figure 6:**
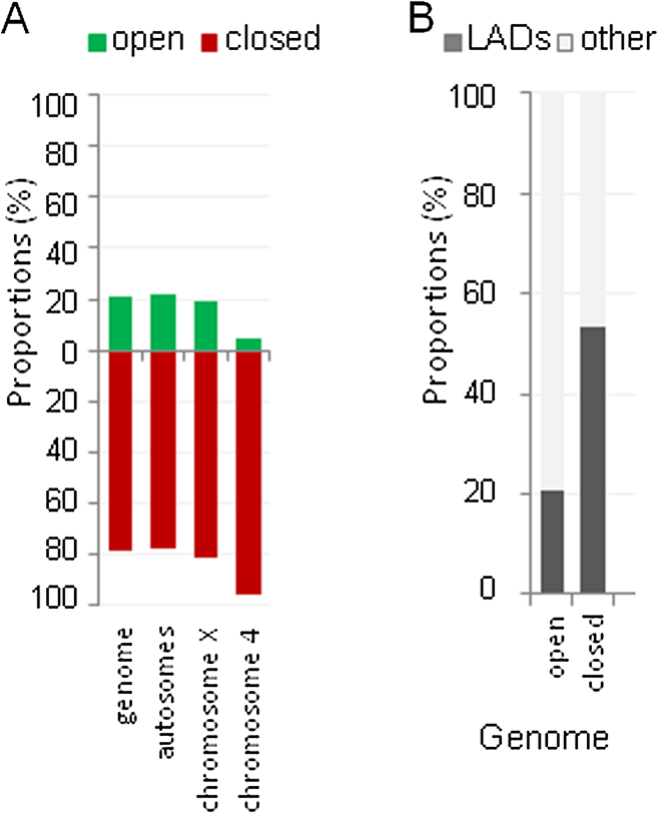
**Link between lamina-associated domains (LADs)**[14]**and closed chromatin detected by 2CM. (A)**, Proportions of closed and open chromatin found in LADs in the entire genome and in its compartments including major autosomes, chromosome X, and chromosome 4. **(B)**, contribution of LADs to the closed and open chromatin in the genome.

Taking into account that morphologically dense heterochromatin is often situated at nuclear periphery, we proposed that a significant proportion of closed chromatin is included in lamina-associated domains (LADs). Indeed, comparison of our GCSDI analysis with the LAD map of *Drosophila* genome [14] revealed that LADs were predominantly closed (Figure 6A, Additional file 7: Figure S7A) and approximately one-half of the closed chromatin in the genome was included in LADs (Figure 6B, Additional file 7: Figure S7B). Considering the emerging major role for lamina in gene repression [15] these findings were consistent with the model in which chromatin compaction is a feature of gene silencing, prompting further inquiry into the relationship between chromatin configuration and gene expression.

**Figure 7:**
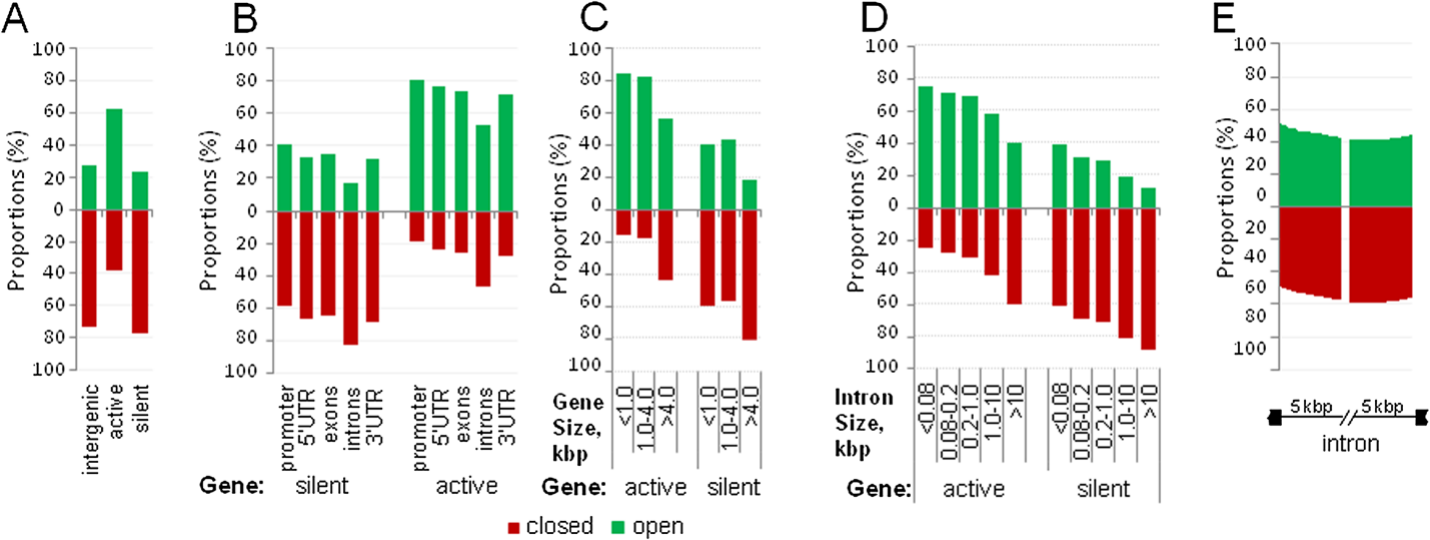
**Relationship between open and closed chromatin and gene structure.** Proportions of open and closed chromatin detected by 2CM are shown for intergenic spacers and active or silent genes **(A)** and for structural elements of active and silent gene **(B)**. Analysis of relationship between chromatin structure and the size of gene **(C)** and intron **(D)** shows that proportion of open chromatin diminishes as the gene and intron size increases for both active and silent genes. **(E)**, Distribution of poen and closed chromatin along large (>10 kbp) active gene introns.

While intergenic spacers were mostly closed or neutral, actively expressed genes were predominantly open and silent genes were generally closed across the genome (Figure 7A,B, Additional file 8: Figure S8A,B). However, this analysis unexpectedly identified a substantial fraction (one-third in 2CM and one-tenth in 3CM) of active gene chromatin in closed configuration. Intriguingly, the gene size appeared a major determinant, with larger active genes displaying more closed chromatin (Figure 7C, Additional file 8: Figure S8C). Structural elements of the active genes were predominantly open with a single exception of introns that were equally represented by the open and closed chromatin, relevant to the aforementioned promiscuous distribution of the predicted chromatin state 4. Interestingly, the proportion of introns with closed chromatin configuration increased rapidly as intron length exceeded 1 kbp (Figure 7D, Additional file 8: Figure S8D). Within the long introns of active genes, the closed chromatin content was the highest in the middle and gradually decreased over several kbp toward the exon/intron borders (Figure 7E, Additional file 8: Figure S8E). Taking into account a rapid transition of RNA polymerase across large introns [16], it can be proposed that chromatin in these regions can quickly condense once the transcription complex has passed. This apparent disconnect between the activity of the genes with large introns and the intron chromatin structure probably underlies regulation of interleaved gene arrangements, where small nested genes often show little correlation with expression of larger including genes that harbor them in introns [17].

**Figure 8:**
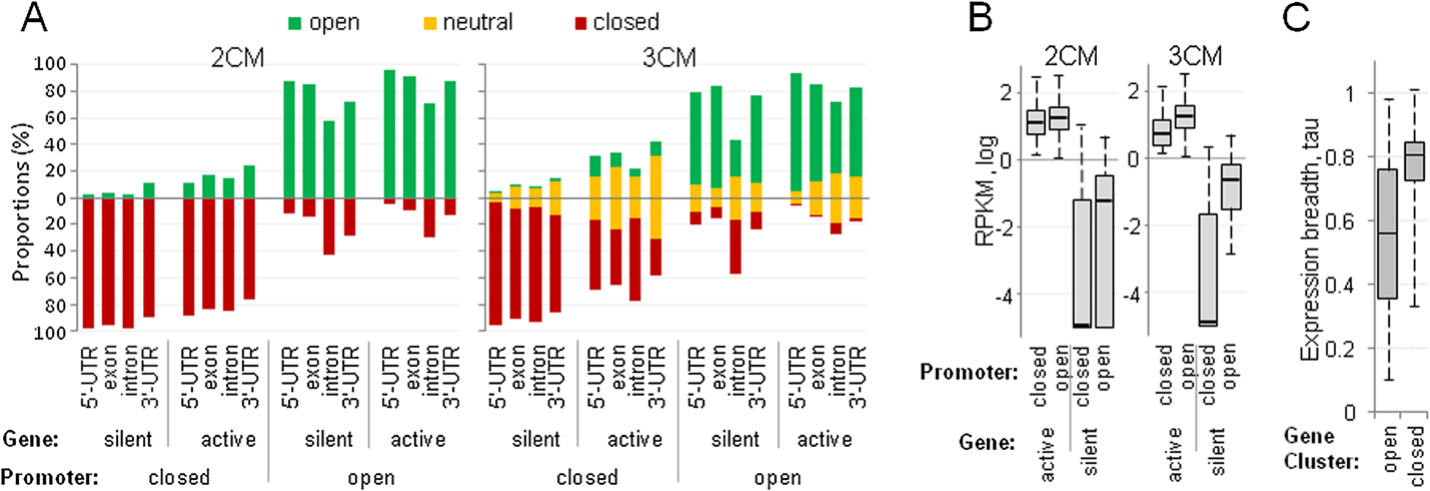
**Chromatin compactness versus gene expression.** Representation of open, closed, and neutral chromatin is shown for structural elements of genes that differ in their expression status and promoter chromatin configuration **(A)**. **(B)**, RNAseq reads per kilobase pair of gene per million (RPKM) are shown as surrogate expression levels for silent and active genes with open or closed promoters. **(C)**, expression breadth metric *tau*[20] of testis-biased genes from gene clusters embedded in open versus closed chromatin domains. Note that if *tau* value is higher, the expression breadth is lower and thus tissue-specificity of gene expression is increased.

Another intriguing finding was the presence of closed chromatin in some active gene promoters (17% in 2CM and 3% in 3CM) and open chromatin in silent gene promoters (one-third in 2CM and 17% in 3CM). We first sought to rule out the trivial explanations such as frequent presence of alternative inactive promoters in active genes, as well as imprecision of chromatin analysis or incorrect selection of the promoter regions. In these cases, even though a promoter may appear in “odd” configuration, the chromatin structure of the gene body would match its expression status. We found just the opposite - the chromatin configuration of the gene body followed that of the promoter (Figure 8A) indicating that some genes can be active in closed chromatin and also that some silent genes are open. The sets of genes defined as active or silent were still clearly distinct in their expression levels regardless of their promoter chromatin configuration, however while silent genes with closed promoters showed essentially no detectable expression at all, a significant fraction of their counterparts with open promoters demonstrated very low but noticeable expression (Figure 8B) consistent with the model in which chromatin compaction completely shuts down expression of silenced genes while opening (“potentiation”) of chromatin exposes genes to transcriptional machinery [1]. Closing of chromatin domains may be used for strict control of tissue-specific genes, especially those organized in large clusters on chromosomes [18, 19]. To test this suggestion, we analyzed 66 clusters of three or more testis-biased genes [19] and found that 28 of them represented uninterrupted domains of closed chromatin, 23 - continuous domains of open chromatin, and only 15 had a transition between open and closed domains within the cluster. We further analyzed cluster genes from the uninterrupted domains of open or closed chromatin. Genes from closed clusters (n = 122) indeed had higher tissue specificity and thus tighter transcriptional control than their counterparts from open clusters (n = 72) as their expression breadth metric *tau*[20] was significantly higher (p < 10^-8^, t-test and U-test) (Figure 8C).

## Conclusions

Intriguingly, we also found that active genes with closed promoters showed lower transcript levels than conventionally expected active gene with open promoters, indicating that chromatin compactness may serve to modulate active gene expression. The mechanisms underlying this type of regulation warrant further inquiry, as do the other unexpected trends identified in our study, such as the general paucity of known chromatin marks positively identifying closed chromatin and the tendency of large introns to stay in closed configuration even when the genes are expressed. We expect that the novel analysis of epigenomic regulation with a straightforward and sensitive assay described here will contribute an empirical approach supplementing predictive chromatin structure assessments, thereby advancing both basic and biomedical research in chromatin biology.

## Methods

### General chromatin sensitivity to DNase I

DNase I treatment of cells was performed as previously described [8] with minor modifications. 1×10^6^ S2 cells were permeabilized with 0.05% NP40 and resuspended in DNase I Buffer (40 mM Tris–HCl, 0.4 mM EDTA, 10 mM MgCl2, 10 mM CaCl2, 0.1 mg/ml BSA). Part of each sample was set aside and later used as non-digested control. DNase I (Promega) digestion was performed at 37°C for 10 or 15 minutes with diverse amounts of the enzyme to optimize the procedure (0.1U, 0.5U, 1U, 2U and 5U); for further analysis cells were treated with 0.5U DNase I for 10 minutes. 20 ng of DNA purified from the treated cells using the DNeasy Blood & Tissue Kit (Qiagen) were used as a template for whole genome amplification. The library preparation step using GenomePlex WGA2 Kit was followed by amplification with the GenomePlex WGA 3 Reamplification Kit (Sigma). dUTP was incorporated at the amplification step to enable the probe fragmentation procedure according to Affymetrix recommendations. The amplification product was purified with the Wizard SV Gel and PCR Clean-Up System (Promega), fragmented and labeled using the GeneChip WT Double-Stranded DNA Labeling Kit (Affymetrix), and hybridized with GeneChip Drosophila Tiling 2.0R Array following the manufacturer’s instructions.

### GCSDI data analysis and genome segmentation

Raw data from microarray CEL files were normalized using CisGenome [21] and log2 differential signals were calculated for each probe.

To generate two-configuration model (2CM), probes with log2 differential signal within 1 standard deviation of the mean were discarded and the signals for the remaining probes were capped at 1 for positive and -1 for negative. A sliding window was used to determine transition points between open (positive) and closed (negative) segments. The difference (d) between the mean log2 differential signals of flanking regions was calculated for every probe. The cutoff d values were established by analyzing 100 permutations of probes, with the requirement that real genome data is significantly different from permuted models (p < 0.05). A series of significant d values and flanking region sizes (n) were tested to determine the model most discriminating between real genome and random permutation. The results presented here are based on analysis using n = 48 and d = 0.8, which identified 2244 transition probes in the real dataset and only 72 on average in permutated controls.

To generate three-configuration model (3CM), all probes were analyzed for presence of differential signal peaks using an HMM algorithm built in CisGenome, with a posterior probability greater than 0.5. An FDR value of 0.1 was used to filter the detected peaks which were further consolidated into domains as following: two adjacent peaks are joined if the distance between them is less that threshold value (16721 bp, which is 95th percentile of inter-peak distances in fly genome), and if they are of the same sign (either both positive or both negative). Otherwise, the segment between the peaks is assigned neutral state. 2CM and 3CM domain coordinates in BDGP5 genome annotation are provided as tables in Additional files 9 and 10.

### Association of segments with lamina-associated domains (LADs), histone modifications, and chromatin states

LAD coordinates [14] were downloaded from NCBI GEO (GSE20313). The coordinates for both “Binding Sites” and “Depleted” regions for 49 individual histone modifications were obtained from the modENCODE project (http://www.modencode.org) [22]; specifically, the following datasets were used: H1.S2; H2AV_9751.S2; H2BK5ac.S2; H2B.ubiq.NRO3..S2; H3 antibody2.S2; H3K9ac.S2; H3K18ac.S2; H3K23ac.S2; H3K27Ac.S2; H3K27me1.S2; H3K27me2_TJ.S2; H3K27me3.Abcam2..S2; H3K36me1.S2; H3K36me3.S2; H3K4me1.S2; H3K4me2.ab.S2; H3K4Me2.Millipore.S2; H3K4me3_S2; H3K79me1.S2; H3K79Me2.S2; H3K79Me.S2; H3K9acS10P_.new_lot..S2; H3K9ac.S2; H3K9me1_Diagenode.S2; H3K9me1.S2; H3K9me2.Ab2.new_lot.S2; H3K9me2Antibody2.S2; H3K9me3.S2; K3K9me3_clone_6F12_H4S2; H4.S2; H4K5ac.S2; H4K8ac.S2; H4K12ac.S2; H4K16ac(L).S2; H4K16ac(M).S2; H4AcTetra.S2; H4K20me.S2; Hp1a_552.S2; HP1a_hinge.S2; HP1a_wa184.S2; HP1a_wa191.S2. DHS data were obtained from the on-line resource http://compbio.med.harvard.edu/flychromatin/data.html[4]. Coordinates for the 9 predicted chromatin states were obtained from the Modencode progect [4]. All datasets were converted to the BDGP5 Drosophila genome annotation as needed. In order to determine the association of chromatin compactness and each of the above genome annotations, we calculated the cumulative overlap between the open, closed, and neutral segments and the previously characterized LADs, histone modification enriched/depleted regions, and predicted chromatin states. The analyses were performed for whole genome as well as for individual chromosomes and their heterochromatic compartments.

### Association of segments with gene expression

We downloaded short-read (Illumina) sequences for 5 massively parallel mRNA sequencing experiments on S2 cells from two GEO datasets (GSM390063 and GSM390064), aligned these reads to the Drosophila reference genome (BDGP5) using TopHat [23], and calculated the Reads Per Kilobase of transcript per Million mapped reads (RPKM) for each gene reported in the BDGP5 reference annotation. We used the criteria of genes with an RPKM value of at least 1 in all five samples to classify genes as either active or inactive. The cumulative overlaps of open, closed, and neutral segments with active and inactive genes were computed to determine the association of chromatin compactness with gene expression. Similar analysis was conducted to determine associations with gene length and different functional regions of a gene (promoter, exon, intron, 5′UTR and 3′UTR regions). To analyze the link between gene/intron size and chromatin structure, the genes were separated into three categories (less than 1 kb, 1–4 kb, and more than 4 kb) and the introns were separated into 5 categories (81 bp or less, 81–200 bp, 201 bp – 1 kb, 1–10 kb, and larger than 10 kb). Cumulative overlaps of these categories of genes and introns with open, closed, and neutral segments were computed. In order to determine the profile of chromatin compactness within introns larger than 10 kb, the introns were divided into non-overlapping windows of 100 bp. These windows were pooled from all introns according to their position relative to the 5′ and 3′ intron ends, and the cumulative overlaps with chromatin compactness segments were computed. The ratio of the open to close states was derived separately for the long medium intron (1 kb – 10 kb) and long intron (>10 kb) to illustrate the transition of DNAse I hypersensitivity states within intronic regions of active and inactive genes.

## Electronic supplementary material

Additional file 1: Figure S1: Evaluation of the amplified DNA from DNase I-treated and untreated cells. Low amplification bias evident by the absence of discrete bands in agarose gel (A), and selective depletion of open chromatin in sample from cells treated with DNase I under diverse conditions (B, C, qPCR data normalized to untreated control, error bars indicate standard error of the mean). Four genome regions with known chromatin compactness were analyzed: *actin* and *Letm1* representing open chromatin, and *Crtp* and *Yu* representing closed chromatin [8]. (JPEG 43 KB)

Additional file 2: Figure S2: Length of chromatin domains detected by 2CM and 3CM models. Length distributions for domains of open, closed, and neutral chromatin shown for the entire size range (right panels) and in more detail for the lower size ends of distribution histograms (left panels). (JPEG 59 KB)

Additional file 3: Figure S3: Distribution of open (green), neutral (yellow), and closed (red) chromatin domains detected by 3CM analysis on chromosomes of *D. melanogaster*. (JPEG 44 KB)

Additional file 4: Figure S4: Representation of detected open ,neutral, and closed chromatin domains in genome and their association with predicted chromatin states. Proportions of open and closed chromatin detected by 3CM are shown for individual chromosomes (A) and for the genome regions predicted as 9 chromatin states [4] (B). 2CM and 3CM analyses also shown for the four sub-states which comprise the predicted state 4 [4] (C). (JPEG 41 KB)

Additional file 5: Figure S5: Contributions of the predicted chromatin states to open, neutral, and closed chromatin detected by 3CM. Results are shown for the whole genome, and separately for major autosomes and chromosomes X and 4. (JPEG 49 KB)

Additional file 6: Figure S6: Enrichment and depletion of chromatin modifications in open, neutral, and closed chromatin. Heat maps show percent proportions of regions enriched with (red) or depleted of (blue) particular chromatin modifications in open and closed chromatin domains detected by 2CM and 3CM. Data are cumulative for the entire genome, euchromatin of major autosomes and chromosome X, the entire chromosome 4, and combined pericentromeric heterochromatin of major autosomes and chromosome X. (JPEG 104 KB)

Additional file 7: Figure S7: Link between lamina-associated domains (LADs) [14] and closed chromatin. Proportions of closed, open, and neutral chromatin detected by 3CM in LADs (A) and contribution of LADs to the closed, open, and neutral chromatin detected by 2CM and 3CM (B) are shown for the entire genome and in its compartments including major autosomes, chromosome X, and chromosome 4. (JPEG 45 KB)

Additional file 8: Figure S8: Relationship between open and closed chromatin and gene structure. Proportions of open, closed, and neutral chromatin detected by 3CM are shown for intergenic spacers and active or silent genes (A) and for structural elements of active and silent gene (B). Analysis of relationship between chromatin structure and the size of gene (C) and intron (D) shows that proportion of open chromatin diminishes as the gene and intron size increases for both active and silent genes. (E), Distribution of open and closed chromatin along large (>10 kbp) active gene introns. (JPEG 58 KB)

Additional file 9: **GCSDI domains 2CM.** (XLSX 64 KB)

Additional file 10: **GCSDI domains 3CM.** (XLSX 645 KB)
