# Supplementary figures and images for: Map of open and closed chromatin domains in Drosophila genome
Source: BMC Genomics. 2014 Nov 18;15(1):988. doi: 10.1186/1471-2164-15-988 (PMC4289254; doi:10.1186/1471-2164-15-988)

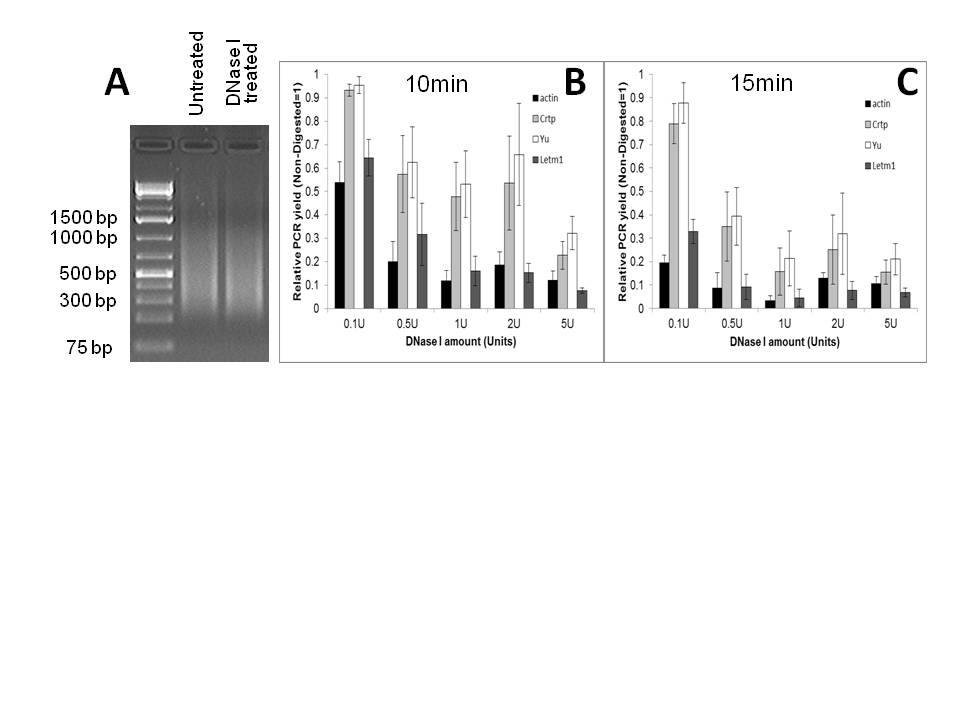

Supplement: Supplementary file 1 — Additional file 1: Figure S1: Evaluation of the amplified DNA from DNase I-treated and untreated cells. Low amplification bias evident by the absence of discrete bands in agarose gel (A), and selective depletion of open chromatin in sample from cells treated with DNase I under diverse conditions (B, C, qPCR data normalized to untreated control, error bars indicate standard error of the mean). Four genome regions with known chromatin compactness were analyzed: actin and Letm1 representing open chromatin, and Crtp and Yu representing closed chromatin [8]. (JPEG 43 KB) [file 12864_2014_6860_MOESM1_ESM.jpeg]

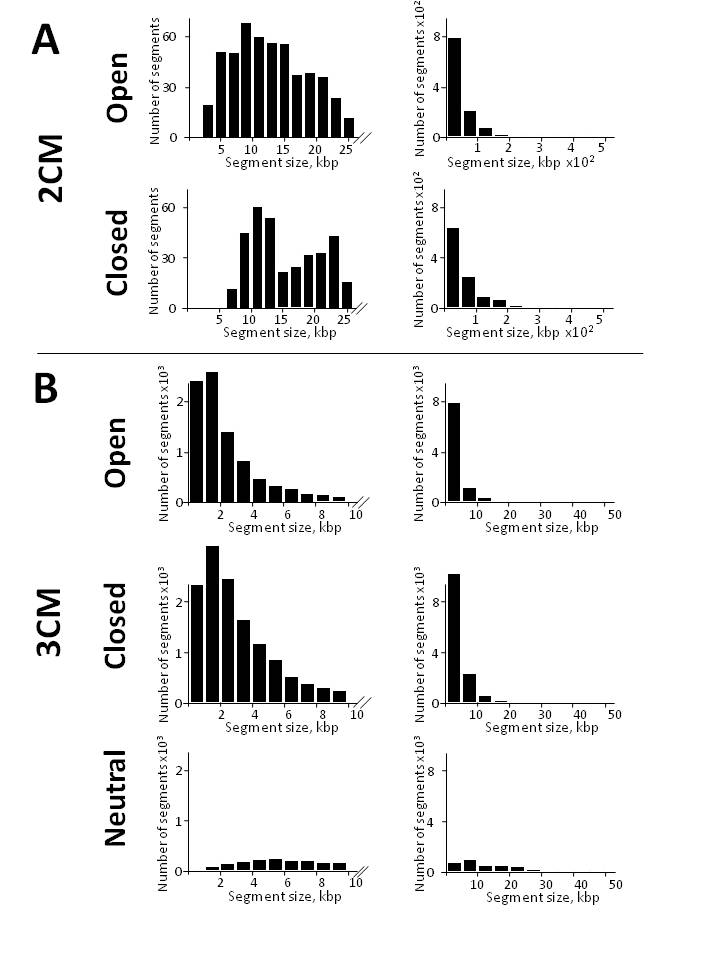

Supplement: Supplementary file 2 — Additional file 2: Figure S2: Length of chromatin domains detected by 2CM and 3CM models. Length distributions for domains of open, closed, and neutral chromatin shown for the entire size range (right panels) and in more detail for the lower size ends of distribution histograms (left panels). (JPEG 59 KB) [file 12864_2014_6860_MOESM2_ESM.jpeg]

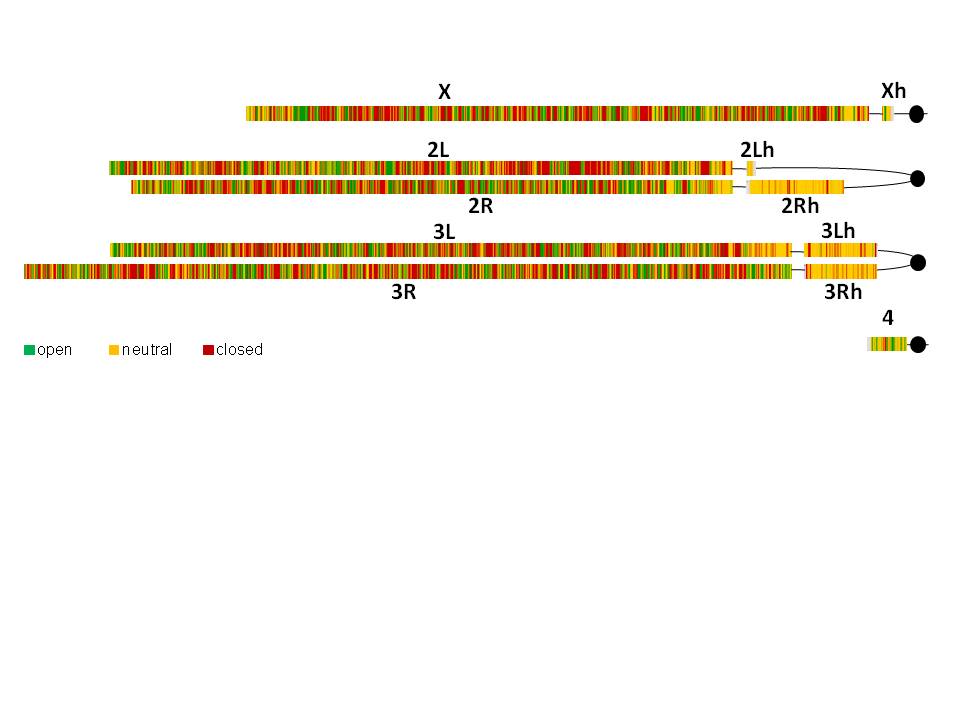

Supplement: Supplementary file 3 — Additional file 3: Figure S3: Distribution of open (green), neutral (yellow), and closed (red) chromatin domains detected by 3CM analysis on chromosomes of D. melanogaster. (JPEG 44 KB) [file 12864_2014_6860_MOESM3_ESM.jpeg]

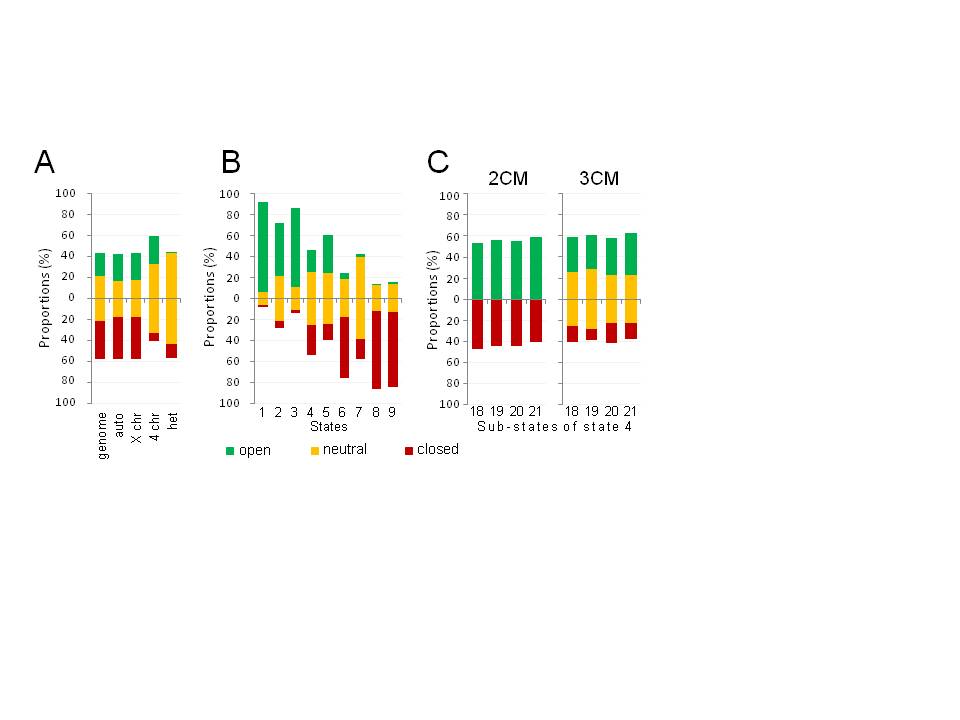

Supplement: Supplementary file 4 — Additional file 4: Figure S4: Representation of detected open ,neutral, and closed chromatin domains in genome and their association with predicted chromatin states. Proportions of open and closed chromatin detected by 3CM are shown for individual chromosomes (A) and for the genome regions predicted as 9 chromatin states [4] (B). 2CM and 3CM analyses also shown for the four sub-states which comprise the predicted state 4 [4] (C). (JPEG 41 KB) [file 12864_2014_6860_MOESM4_ESM.jpeg]

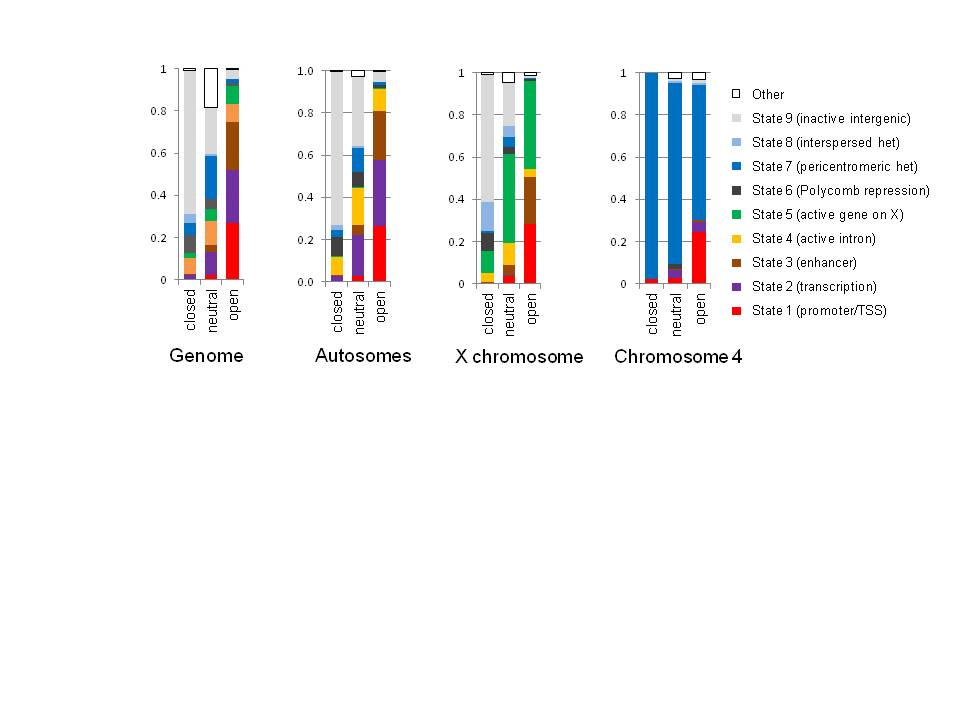

Supplement: Supplementary file 5 — Additional file 5: Figure S5: Contributions of the predicted chromatin states to open, neutral, and closed chromatin detected by 3CM. Results are shown for the whole genome, and separately for major autosomes and chromosomes X and 4. (JPEG 49 KB) [file 12864_2014_6860_MOESM5_ESM.jpeg]

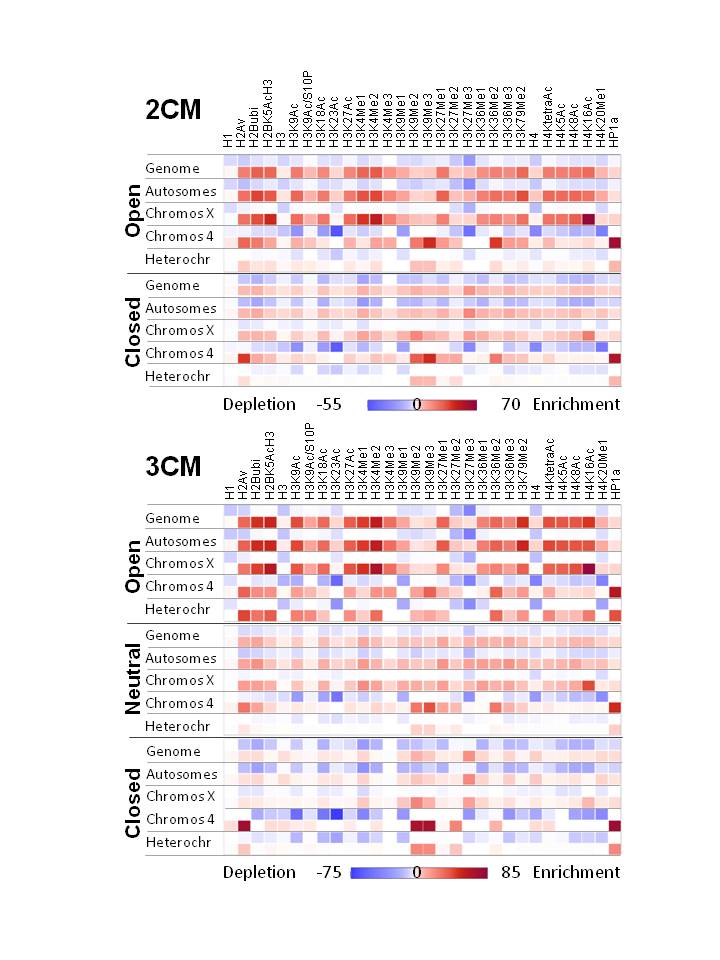

Supplement: Supplementary file 6 — Additional file 6: Figure S6: Enrichment and depletion of chromatin modifications in open, neutral, and closed chromatin. Heat maps show percent proportions of regions enriched with (red) or depleted of (blue) particular chromatin modifications in open and closed chromatin domains detected by 2CM and 3CM. Data are cumulative for the entire genome, euchromatin of major autosomes and chromosome X, the entire chromosome 4, and combined pericentromeric heterochromatin of major autosomes and chromosome X. (JPEG 104 KB) [file 12864_2014_6860_MOESM6_ESM.jpeg]

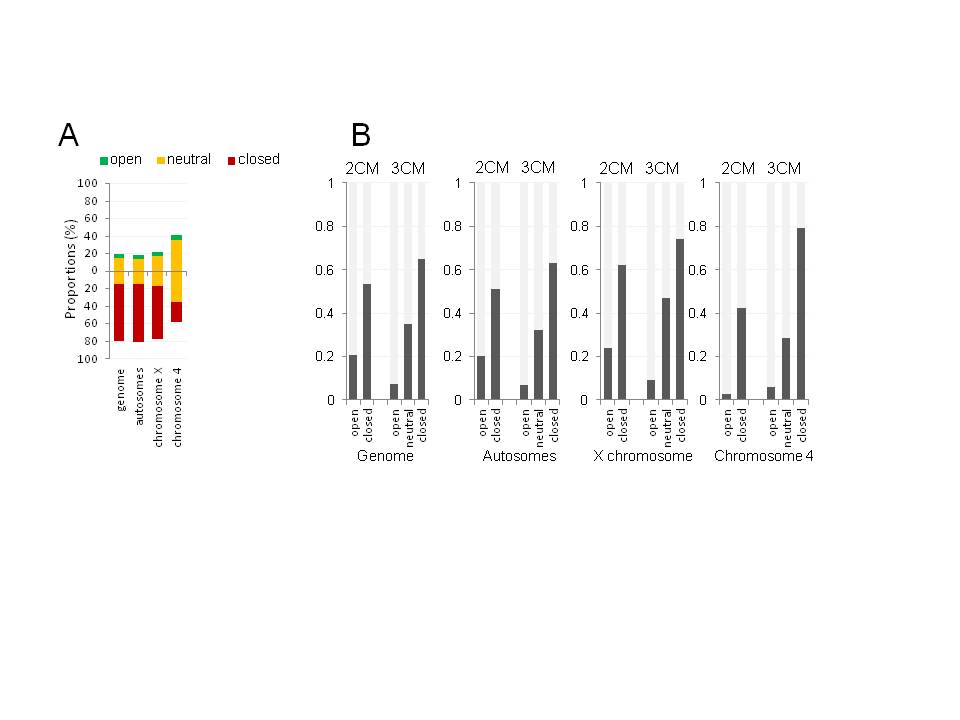

Supplement: Supplementary file 7 — Additional file 7: Figure S7: Link between lamina-associated domains (LADs) [14] and closed chromatin. Proportions of closed, open, and neutral chromatin detected by 3CM in LADs (A) and contribution of LADs to the closed, open, and neutral chromatin detected by 2CM and 3CM (B) are shown for the entire genome and in its compartments including major autosomes, chromosome X, and chromosome 4. (JPEG 45 KB) [file 12864_2014_6860_MOESM7_ESM.jpeg]

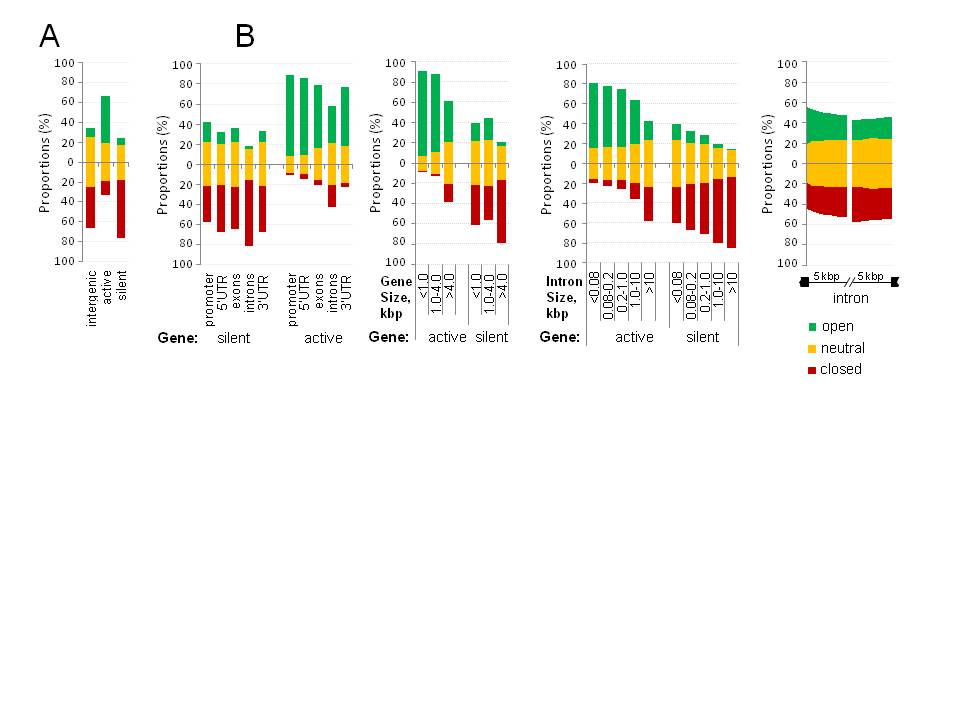

Supplement: Supplementary file 8 — Additional file 8: Figure S8: Relationship between open and closed chromatin and gene structure. Proportions of open, closed, and neutral chromatin detected by 3CM are shown for intergenic spacers and active or silent genes (A) and for structural elements of active and silent gene (B). Analysis of relationship between chromatin structure and the size of gene (C) and intron (D) shows that proportion of open chromatin diminishes as the gene and intron size increases for both active and silent genes. (E), Distribution of open and closed chromatin along large (>10 kbp) active gene introns. (JPEG 58 KB) [file 12864_2014_6860_MOESM8_ESM.jpeg]
